# Using Google Trends to Estimate the Geographic Distribution of Soil-Transmitted Helminthiasis in the United States from 2016 to 2021

**DOI:** 10.3390/tropicalmed8040212

**Published:** 2023-04-01

**Authors:** Steven H. Adams, Timothy P. Endy, David A. Larsen

**Affiliations:** 1 Department of Pathology, Stony Brook University Hospital, Stony Brook, NY 11794, USA; 2 Departments of Internal Medicine and Infectious Diseases, SUNY Upstate Medical University, Syracuse, NY 13210, USA; 3 Department of Public Health, Syracuse University, Syracuse, NY 13244, USA

**Keywords:** Google Trends, hookworm, roundworm, USA

## Abstract

Soil-transmitted helminth infections are assumed to be uncommon in the US, despite numerous studies in the past few decades showing high burdens in Appalachia and the southern states. We assessed trends of interest in the Google search engine to gauge spatiotemporal patterns of potential soil-transmitted helminth transmission. We conducted a further ecological study comparing Google search trends to risk factors for soil-transmitted helminth transmission. Google search trends for terms related to soil-transmitted helminths were clustered in Appalachia and the south, with seasonal surges suggestive of endemic transmission for hookworm, roundworm (Ascaris), and threadworm. Furthermore, lower access to plumbing, increased septic tank use, and more rural environments were associated with increased soil-transmitted helminth-related Google search terms. Together, these results suggest that soil-transmitted helminthiasis remains endemic in parts of Appalachia and the south.

## 1. Introduction

Soil-transmitted helminths are parasitic worms that infect the gastrointestinal tract and have an infective life stage in soil. Early symptoms include abdominal pain and abdominal bloating. Transmission occurs when either humans ingest helminth eggs through contaminated food or hand-to-mouth behavior, or for the case of hookworm, when infective larvae penetrate the skin of the foot (or are ingested). Upon penetrating the skin of the foot, hookworm larvae can cause localized irritation including a rash and itch. Eggs are shed in human feces, and as such improving sanitation access (toilets and hand washing) effectively reduces and can even interrupt transmission. The number of disability-adjusted life years lost globally due to soil-transmitted helminths was estimated to have decreased by 53% from over 4 million in the year 2000 to 1.9 million in the year 2019 due primarily to increased access to deworming medicines [1]. The bulk of disability-adjusted life years lost occur in lower-income countries in communities with poor access to water, sanitation, and hygiene [2]. It is generally assumed that soil-transmitted helminthiasis (STH) is uncommon in the contemporary United States.

The assumption that the transmission of STH has been eliminated in the US was reinforced by a 2011 review [3]. More recent reports suggest that transmission is probable in distinct populations of the country [4]. For example, *Strongyloides stercoralis* infections were found to be common in a low-income community in Texas [5], hookworm infections were common in Lowndes County, Alabama [6]; intestinal inflammation was associated with STH in Mississippi [7]; and STH was recently found throughout soil samples in the south [8]. The sporadic nature of reports of STH in the US are indicative of either sporadic transmission (perhaps due to travelers returning from endemic countries) or gaps in the infectious disease surveillance system allowing the endemic transmission of STH to go unnoticed. Traditional infectious disease surveillance systems rely on case diagnosis. Unfortunately, the people at the highest risk of STH are also the people with the lowest access to health care. Furthermore, if clinicians are not actively testing for STH, then cases of STH will not be found.

To increase the understanding of potential endemic STH transmission in the United States, we utilized search engine activity, specifically Google Trends (GT). In recent years, researchers have shown that Internet search data correlate well with infectious disease transmission, even forecasting outbreaks of various infectious diseases including influenza, varicella, dengue fever, and chikungunya [9,10,11,12]. More recently, Google engine interest in COVID-19-related search terms has been shown to predict COVID-19 outbreaks using Trends [13]. Additionally, while individuals at high risk of STH may have lower access to health care, these individuals typically have access to a Google search engine—often through a telephone. Using GT, we estimate the relative geographic distribution of the STH burden for the years 2016–2021 as reflected in the search volume traffic, focusing on the soil-transmitted helminths, *Ancylostoma duodenale* and *Necator americanus* (hookworms), *Ascaris Lumbricoides* (giant roundworms), *Trichuris trichiura* (whipworms), and *S. stercoralis* (threadworms) species which have had a historical presence in the United States [3,4]. Although by all definitions they are soil-transmitted helminth, we have not included specific search terms for the zoonotic *Toxocara canis* and *T. cati* in our analyses [4,14].

## 2. Methods

### 2.1. Google Trends

We utilized the Google Trends application (https://trends.google.com/trends/?geo=US accessed on 7 May 2022) to collect data on STH-related Google searches in the USA. Data are provided by Trends as a relative search volume index (RSV) which standardizes the popularity of a search term relative to the total number of searches over a selected period of time in a specific geographical area. The RSV score is scaled on a range of 0 to 100 such that the peak search volume point receives a score of 100. Trends report a 0 if the total searches for a specific term do not surpass an unreported privacy threshold which is based upon absolute numbers of searches within the timeframe and geographic location selected [15]. Both regional and temporal (monthly) search volume data are supplied. Google makes its Trends data available for download for further analysis. The sequence of words in a user’s search is not significant, as Trends includes results for any order [15]. Duplicate searches for the same term by one searcher are excluded as GT eliminates repeated queries from the same user conducted over a short timeframe [16]. GT also sets an unreported privacy threshold, not reporting RSV when the absolute number of searches falls below a specific threshold.

### 2.2. Selection of Search Terms

In the GT application, searches were limited to the “United States” geographical region, occurring between 1 January 2016 and 29 December 2021. Other inputs included “all categories”, in category selection, and “web search” for modality. Searches were limited to 2016–2021 due to changes made to Trends’ collection system prior to 2016 [17]. Terms that people suffering from STH may search for online were chosen, such as stool worms, poop worms, hookworms, and roundworms, as well as names of various anthelmintic medications. Both singular and plural forms of the search terms, as well as combinations of terms with prepositions and conjunctions, were queried. Using this method, 142 STH-related terms were investigated on GT (see Supplementary File S1). After setting a cutoff threshold of ≥50% (search terms with ≥50% of states reporting a value greater than zero), only 56 terms remained (Table 1: 1–11) (≥50% was chosen as the cutoff because we found that terms with a lower yield consistently showed a preference for high-population states such as California, Texas, and Florida).

### 2.3. Grouping of Search Terms

The 56 search terms were then grouped into 13 broad categories as outlined in Table 1 for temporal analyses and Table 2 for spatial analyses. Terms which varied from each other in only minor linguistic nuances such as plural vs. singular form, or with/without a conjunction or preposition, were grouped into a single category. Terms with identical meaning but expressed in the vernacular vs. scientific nomenclature were grouped separately (e.g., “roundworm” and “*Ascaris lumbricoides*” were separate categories, but “roundworm” and “roundworms” were a single category). The 15 sanitation-infrastructure-deficiency-related search terms were grouped into a single category. The mean of each individual search term within these broad search term categories was then used as the outcome measure [1,2,3,4].

### 2.4. Spatial Analysis of National Searches

Spatial clustering in search term categories by state was examined using the global Moran I test. We visualized search intensities using the ggplot2 and urbnmapr packages, and tested for clustering using the spdep package in R version 4.0.3 [18,19,20,21]. We then assessed the relationship by state between STH-related search term intensity and (a) sanitation-infrastructure-deficiency-related search term intensity, and (b) factors of state levels of median income, unemployment, percent of population living in rural areas, average temperature, plumbing access, and septic tank use, employing an unadjusted Pearson correlation coefficient.

### 2.5. Temporal Analysis of National Trends

We examined the temporal trends in search term categories with monthly estimates of national level RSV from 2016 to 2021, testing both for seasonality and change over time. Seasons were categorized as winter (December–February), spring (March–May), summer (June–August), and fall (September–November). We visualized time series with Loess smoothing and a span of 0.25, and then utilized linear regression to determine the influence of seasonality and change over time [22]. We tested for serial autocorrelation of residuals using the simple linear model, and if found, we applied a generalized least squares regression with standard errors adjusted for autocorrelation. In the regression analysis of the hookworm category, three outlying observations that coincided with the release of a dramatic film titled “Hookworm” were excluded. We used the ggplot2 and nlme packages in R version 4.0.3 for these analyses [18,21,23].

## 3. Results

### 3.1. Spatial Analysis of National Searches

**Figure 1:**
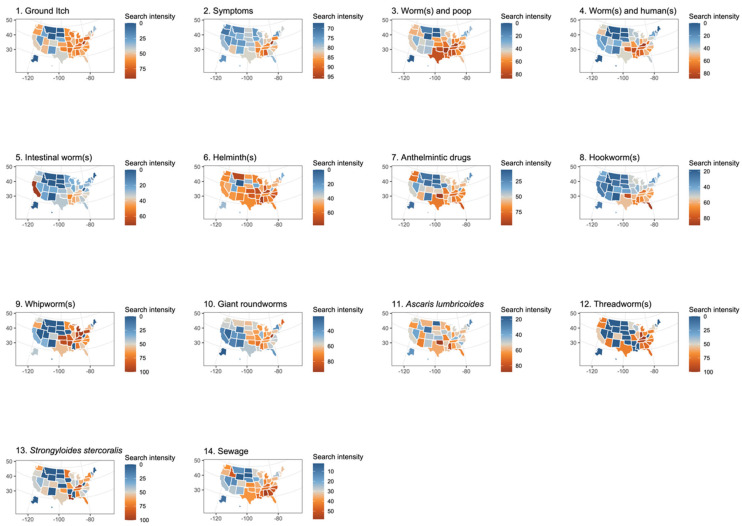
(1–13) Thirteen categories of search terms related to soil-transmitted helminths plotted by state using the mean of data from 2016 to 2021. (14) Category of search terms related to sanitation infrastructure deficiencies plotted by state using the mean of data from 2016 to 2021. The number for each map corresponds to Table 2, categories 1–14, showing the exact search terms used.

Visualizing the search engine traffic suggests a clustering of searches in Appalachia and the south (Figure 1), with significant clustering in every search term with the exception of the *S. stercoralis* category (cat. 13). Increased search traffic among all search term categories was associated with increased search traffic of sanitation infrastructure deficiency terms (cat. 14, Table 3). At the state level, limited associations were observed between potential factors (Table 3, items 1–6) and search term intensities. Lower state-level income was associated with increased search traffic for ten categories, namely “ground itch” (cat. 1), symptoms (cat. 2), “worms and poop” (cat. 3), “worms and humans” (cat. 4), “intestinal worms” (cat. 5), helminths (cat. 6), hookworms (cat. 8), roundworms (cat. 10), *Ascaris* (cat. 11), and sanitation infrastructure deficiency (cat. 14) terms. Lower unemployment levels were associated with increased searches for roundworms (cat. 10). Lower access to plumbing was associated with increased searches for “ground itch” (cat. 1) and “worms and poop” (cat. 3). More houses with septic tanks were associated with increased searches for symptoms (cat. 2), hookworms (cat. 8), roundworms (cat. 10), and sanitation infrastructure deficiency (cat. 14) terms. Increased temperature was associated with increased searches for 11 categories, namely “ground itch” (cat. 1), symptoms (cat. 2), “worms and poop” (cat. 3), worms and humans (cat. 4), intestinal worms (cat. 5), helminths (cat. 6), anthelmintic drugs (cat. 7), hookworms (cat. 8), *A. lumbricoides* (cat. 11), *S. stercoralis* (cat. 13), and sanitation infrastructure deficiency (cat. 14). States with more rural populations were more likely to search for *Ascaris* (cat. 11), *S. stercoralis* (cat. 13), and sanitation infrastructure deficiency (cat. 14).

### 3.2. Temporal Analysis of National Trends

**Figure 2:**
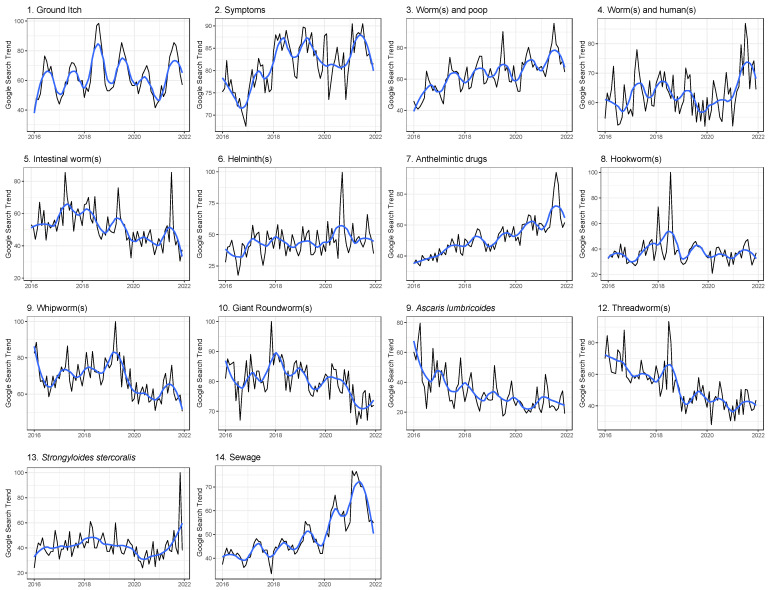
Trends in monthly search engine traffic for fourteen categories of searches related to soil-transmitted helminths from 2016 to 2021. The black line shows the actual measure of search engine traffic. The blue line shows a Loess smoothing with a span limited to 0.25. (1–13) Thirteen categories of search terms related to soil-transmitted helminths plotted by state using the mean of data from 2016 to 2021. (14) Category of search terms related to sanitation infrastructure deficiencies plotted by state using the mean of data from 2016 to 2021. The number for each map corresponds to Table 1, categories 1–14, showing the exact search terms used.

Visualizing the search engine traffic suggests a seasonality to searches related to STH across the United States (Figure 2), with some suggestion that search engine traffic increased over the time period. As seen via regression analysis (Table 4), seasonality was not consistent across search term categories, however, with “ground itch” (cat. 1), “worms and poop” (cat. 3), intestinal worms (cat. 5), anthelmintic(s) (cat. 7), hookworms (cat. 8), and sanitation infrastructure deficiency (cat. 14) having higher searches in the summer months, but helminths (cat. 6), roundworms (cat. 10), and *A. lumbricoides* (cat. 11) having higher searches in the winter months. Increasing yearly search traffic over time was also not consistent across search term categories, with the categories of symptoms (cat. 2), “worms and poop” (cat. 3), anthelmintic(s) (cat. 7), and sanitation infrastructure deficiency increasing (cat. 14), but the categories of whipworms (cat. 9), *A. lumbricoides* (cat. 11), and threadworms decreasing over the time period. Trends in sanitation infrastructure deficiency (cat. 14) were associated with the terms “ground itch” (cat. 1), symptoms (cat. 2), worms and humans (cat. 4), and helminths (cat. 6), but not with any other categories.

## 4. Discussion

Our findings related to Google search traffic indicate that STH infections may continue to persist in distinct regions of the United States. Historically, STH infections in the United States were common throughout Appalachia and the American South [3]. Our spatial analyses show that Appalachia and the American South had higher Google search traffic for all things related to STH infection. For example, Kentucky, a state with uniquely increased history of endemic strongyloidiasis as compared to other states [24,25,26,27], shows the strongest search intensity of all of the states for both threadworms and *S. stercoralis* (Figure 1). Consistent findings of seasonal patterns within the Google search traffic data are further indicative of search traffic volume reflecting infectious disease symptoms in real time. Seasonal analysis revealed increased search traffic for numerous categories in the summer, when we expect transmission to be the most common.

The associations observed between potential risk factors of STH transmission and STH search term intensities are more ambivalent. While one indicator of poverty (median income) was positively correlated with STH-related search traffic, another indicator (unemployment) was inversely correlated. Occupational exposure through agriculture or working with children might explain this discrepancy. Increased rural population was only associated with increased searches for roundworms. Unlike city inhabitants who have the benefit of being connected to their cities’ sewer grids, rural dwellers often rely on backyard septic tanks which frequently fall into disrepair creating an environment ideal for STH. Poorer rural areas may employ the “straight piping” method which utilizes crude piping and open pits to direct human excrement away from the home [6,28,29]. Rural living itself, however, does not necessarily suggest poverty or poor sanitation. As expected, an increased average temperature, suggestive of a warmer climate friendly to the helminth life cycle, correlated with an increased search volume for much of the STH-related terms. Access to complete plumbing facilities had minimal associations. However, in 2016–2019, this census question no longer asked about a flush toilet, an integral component of sanitation for the control of STH, and therefore responses are of limited value for measuring STH risk. Though septic tank use was only associated with increased searches for limited search categories, we should keep in mind that (a) the tank data are dated (1990) and likely inaccurately reflect current status, and (b) the data do not reveal whether or not the septic tanks are failing.

These are ecological analyses, and while aligning with various studies suggesting an increase in STH transmission and risk in the United States [4], conclusions are still limited. The use of Google search traffic in research has been criticized due to the lack of full transparency from Google Inc. on how its Trends algorithms are computed [30]. However, Google has not reported any changes to its data collection system for Trends’ algorithm during the period included in our study, and thus the algorithm has remained consistent over this timeframe. This is in contrast to the dates of 1 January 2011 and 1 January 2016, dates whereby Trends notes that improvements to its data collection systems were applied [31]. Furthermore, there is no standardized procedure for search term selection in conducting Internet search-traffic-based research. Other infectious disease Trends studies have limited themselves to only several terms, using the names of diseases of interest alone or with the addition of the word “symptoms” and the like [10,11]. We aimed to include a broader and more inclusive dataset of STH-related search terms in our study, and therefore queried 142 STH-related terms. Lastly, our results only reflect those that sought information on soil-transmitted helminths online through the Google Search engine in the United States and in the English language. During the period selected for investigation, Google claimed an average of 87% of desktop, mobile, and tablet search engine market share in the United States [32].

An inherent limitation to the use of Google Trends in research is that the intentions of the individual searcher are unknown. Searches unrelated to STH infection act as confounders, and certainly searches performed solely out of curiosity or even academic interest act as confounders. Furthermore, people could be searching Google for symptoms related to their pets or farm animals—many of the terms we have used are not specific to humans. People could also be searching Google because they suffer from delusional parasitosis [33], which would be unrelated to STH transmission. A number of examples from our research illustrate these limitations. First, the search term category *S. stercoralis* was the only search term category that showed no clustering. This may be explained by a recent news media article about Strongyloides in The Guardian [34], which has a broad readership. Curiosity-driven searches related to news media coverage would wash out any spatial clustering of searches related to STH infections. Even more complicated is that local news media coverage (or a local awareness) of STH might drive searches in endemic areas and increase Trends results. This phenomenon would not bias the spatial analyses, but would definitely introduce bias into the temporal analyses. Second, several of the terms selected for this study have veterinary functions and thus likely include false positives. Albendazole and ivermectin are also used for the treatment of cattle, sheep, and goats, while pyrantel is a popular canine dewormer [35,36]. We also observed a spike in searches for “ivermectin” in 2021, that is likely related to misinformation from the COVID-19 pandemic. Third, some queries for threadworm may have intended the pinworm, *Enterobius vermicularis* (United Kingdom vernacular usage), rather than *S. stercoralis* [37]. Fourth, a drama film named *Hookworm* was released in 2017 and there is a rock band by the same name [38,39]. On 2 February 2018, the British rock band Hookworms released a new album which quickly achieved popular acclaim [40]. The week of the album release coincides with an abrupt peak in Trends searches for “hookworm” and “hookworms,” apparently reflecting interest in the rock band rather than in helminths. However, a practical motivation behind Google searches can be assumed for terms such as “worms in my poop”, due to the personal and less scholarly nature of such phrases, as compared to “ascariasis”. It was for this reason that we chose to categorize terms expressed in the vernacular vs. scientific nomenclature separately (e.g., “roundworm” and “*Ascaris lumbricoides*” were separate categories). Mimickers of worms in stool, such as mucus cords, vegetable matter, and chewing gum could also yield false positive search results [41].

## 5. Conclusions

Our results suggest that STH transmission remains endemic in parts of Appalachia and the south in a pattern similar to 20th century reports. These findings may serve to increase awareness amongst researchers and physicians in endemic areas as to the prevalence of helminthiasis. Additionally, the findings may assist government bodies in targeting areas that need improvements in wastewater infrastructure. This study further supports the growing body of literature demonstrating GT as being a useful tool for identifying hotspots for emerging infectious diseases.

## Figures and Tables

**Table 1:** Categorization of search terms for temporal analyses.

Category	Search Terms
1. Ground itch	“itchy foot”, “rash on foot”
2. Symptoms	“blood in stool”, “stomach pain”, “bloating”, “anemia”
3. Worms and poop	“poop worm”, “poop worms”, “worms and poop”, “poop with worms”, “worm in poop”, “worms in poop”, “worm in my poop”, “worms in my poop”, “worms in the poop”
4. Worms and humans	“human worm”, “human worms”, “humans worm”, “humans worms”, “human and worm”, “human and worms”, “humans and worm”, “humans and worms”, “worm in human”, “worm in humans”, “worms in human”, “worms in humans”
5. Intestinal worms	“intestinal worm”, “intestinal worms”
6. Helminths	“helminth”, “helminths”
7. Anthelmintic(s)	“Soolantra”, “Sklice”, “pyrantel pamoate”, “mebendazole”, “praziquantel”, “Ivermectin”, “Pyrantel”, “Albendazole”
8. Hookworms	“hookworm”, “hookworms”, “hookworm humans”, “hookworms humans”, “hookworm in humans”, “hookworms in human”, “hookworms in humans”
9. Whipworms	“whipworm”, “whipworms”
10. Giant roundworms	“roundworm”, “roundworms”
11. *Ascaris lumbricoides*	“*Ascaris*”, “ascariasis”, “*Ascaris lumbricoides*”
12. Threadworms	“threadworm”, “threadworms”
13. *Strongyloides stercoralis*	“*Strongyloides*”
14. Sanitation infrastructure deficiency	“poop outside”, “cleaning septic tank”, “septic installation”, “septic repair”, “septic tank service”, “sewer repair”, “septic service”, “septic tank pumping”, “septic tank pump”, “septic pump”, “contaminated water”, “boil water”, “Boil water advisory”, “septic cost”, “septic tank cost”

**Table 2:** Categorization of search terms for spatial analyses.

Category	Search Terms
1. Ground itch	“itchy foot”, “rash on foot”
2. Symptoms	“blood in stool”, “stomach pain”, “bloating”, “anemia”
3. Worms and poop	“poop worm”, “poop worms”, “worms in poop”
4. Worms and humans	“human worms”, “humans worms”, “worms in humans”
5. Intestinal worms	“intestinal worms”
6. Helminths	“helminth”, “helminths”
7. Anthelmintic(s)	“Soolantra”, “Sklice”, “pyrantel pamoate”, “mebendazole”, “praziquantel”, “Ivermectin”, “Pyrantel”, “Albendazole”
8. Hookworms	“hookworm”, “hookworms”, “hookworm in humans”
9. Whipworms	“whipworm”
10. Giant roundworms	“roundworm”, “roundworms”
11. *Ascaris lumbricoides*	“*Ascaris*”, “ascariasis”, “*Ascaris lumbricoides*”
12. Threadworms	“threadworms,” threadworm”
13. *Strongyloides stercoralis*	“*Strongyloides*”
14. Sanitation infrastructure deficiency	“poop outside”, “cleaning septic tank”, “septic installation”, “septic repair”, “septic tank service”, “sewer repair”, “septic service”, “septic tank pumping”, “septic tank pump”, “septic pump”, “contaminated water”, “boil water”, “Boil water advisory”, “septic cost”, “septic tank cost”

**Table 3:** Estimate of spatial clustering (global Moran’s I) from state-level intensity of various search terms as well as unadjusted relationships (Pearson’s correlation coefficient with 95% confidence intervals) between search terms and various factors. The number for each outcome corresponds to Table 2 showing the exact search terms used. Items 1–4 and 6 represent percent of the population rather than absolute numbers.

Item #		Ground Itch ^1^	Symptoms ^2^	Worms and Poop ^3^	Worms and Humans ^4^	Intestinal Worms ^5^	Helminths ^6^	Anthelmintic(s) ^7^
	Moran’s I	0.357 ***	0.423 ***	0.598 ***	0.590 ***	0.503 ***	0.241 **	0.371 ***
	Sanitation-infrastructure-deficiency-related search terms ^12^	0.70 *** (0.53–0.82)	0.53 *** (0.30–0.71)	0.80 *** (0.67–0.88)	0.74 *** (0.58–0.84)	0.70 *** (0.52–0.82)	0.55 *** (0.32–0.72)	0.70 *** (0.52–0.82)
1	Income	−0.19 (−0.44–0.09)	−0.55 *** (−0.71–−0.32)	−0.53 *** (−0.70–−0.30)	−0.50 *** (−0.68–−0.26)	−0.34 * (−0.57–−0.08)	−0.31 * (−0.54–−0.03)	−0.16 (−0.42–0.12)
2	Unemployment	0.03 (−0.25–0.30)	0.11 (−0.17–0.37)	0.06 (−0.22–0.33)	0.19 (−0.09–0.45)	0.13 (−0.15–0.39)	0.21 (−0.07–0.46)	0.11 (−0.17–0.37)
3	Occupied housing units lacking complete plumbing facilities	−0.31 * (−0.54–−0.04)	−0.15 (−0.40–0.14)	−0.29 * (−0.53–−0.02)	−0.24 (−0.48–0.04)	−0.26 (−0.50–0.02)	−0.17 (−0.42–0.11)	−0.27 (−0.50–0.01)
4	Septic tank use	0.10 (−0.18–0.37)	0.42 ** (0.16–0.62)	0.19 (−0.09–0.44)	0.16 (−0.12–0.42)	0.03 (−0.24–0.31)	−0.01 (−0.29–0.26)	−0.05 (−0.32–0.23)
5	Temperature	0.35 * (0.09–0.57)	0.32 * (0.04–0.54	0.57 *** (0.35–0.73)	0.48 *** (0.24–0.67)	0.52 **** (0.28–0.69)	0.39 ** (0.13–0.60)	0.60 *** (0.39–0.75)
6	Rural	−0.07 (−0.34–0.21)	0.27 (−0.01–0.51)	0.19 (−0.09–0.44)	0.21 (−0.07–0.46)	0.08 (−0.20–0.35)	0.12 (−0.16–0.38)	−0.05 (−0.32–0.23)
		**Hookworms ^8^**	**Whipworms ^9^**	**Roundworms ^10^**	***A. lumbricoides* ^11^**	**Threadworms ^12^**	***S. stercoralis* ^13^**	**Sanitation infrastructure deficiency ^14^**
	Moran’s I	0.590 ***	0.318 ***	0.372 ***	0.187 *	0.302 ***	−0.026	0.533 ***
	Sanitation-infrastructure-deficiency-related search terms ^12^	0.71 *** (0.55–0.83)	0.60 *** (0.39–0.75)	0.49 *** (0.25–0.68)	0.42 ** (0.16–0.62)	0.56 *** (0.34–0.73)	0.48 *** (0.24–0.67)	Not included
1	Income	−0.37 ** (−0.58–−0.10)	−0.13 (−0.39–0.15)	−0.55 *** (−0.72–−0.33)	−0.34 * (−0.56–−0.07)	0.00 (−0.27–0.28)	0.03 (−0.25–0.30)	−0.45 *** (−0.65–−0.20).
2	Unemployment	0.07 (−0.21–0.34)	0.13 (−0.15–0.39)	−0.29 * (−0.52–−0.01)	0.12 (−0.16–0.38)	0.11 (−0.17–0.38)	0.10 (−0.18–0.37)	0.10 (−0.18–0.36)
3	Occupied housing units lacking complete plumbing facilities	−0.22 (−0.47–0.06)	−0.08 (−0.34–0.20)	−0.27 (−0.50–0.01)	−0.14 (−0.40–0.14)	−0.21 (−0.46–0.07)	−0.25 (−0.49–0.03)	−0.25 (−0.49–0.02)
4	Septic tank use	0.35 * (0.08–0.57)	0.01 (−0.27–0.28)	0.61 *** (0.40–0.76)	−0.14 (−0.40–0.14)	0.01 (−0.27–0.28)	−0.16 (−0.42–0.12)	0.45 ** (0.19–0.64)
5	Temperature	0.52 *** (0.29–0.70)	0.20 (−0.08–0.45)	−0.01 (−0.28–0.27)	0.44 ** (0.19–0.64)	0.27 (−0.00–0.51)	0.30 * (0.03–0.53)	0.41 ** (0.15–0.61)
6	Rural	0.10 (−0.18–0.37)	0.03 (−0.25–0.30)	0.56 *** (0.33–0.72)	0.14 (−0.14–0.40)	−0.13 (−0.39–0.15)	−0.14 (−0.40–0.14)	0.17 (−0.11–0.43)

* = *p* < 0.05, ** = *p* < 0.01, *** = *p* < 0.001.

**Table 4:** Regression coefficients (95% confidence intervals) of relative search traffic across fourteen different categories of search terms from a national time series of Google search terms. The superscript number for each outcome corresponds to Table 1 showing the exact search terms used.

	Ground Itch ^1^	Symptoms ^2^	Worms and poop ^3^	Worms and humans ^4^	Intestinal worms ^5^	Helminths ^6^	Anthelmintic(s) ^7^
N months	72	72	72	72	72	72	72
Sanitation-infrastructure-deficiency-related search terms ^12^	4.6 * (0.8–8.4)	4.4 *** (2.4–6.3)	1.6 (−1.5–4.7)	3.9 * (0.5–7.4)	−0.3 (−5.0–4.3)	7.9 ** (2.2–13.6)	1.0 (−1.0–3.0)
Year	−2.6 (−7.2–1.9)	4.6 ** (1.6–7.6)	3.3 *** (1.6–5.1)	−0.7 (−2.9–1.5)	−2.5 (−5.1–0.1)	−1.4 (−4.9–2.2)	4.2 *** (3.0–5.3)
Season							
Winter	Reference	Reference	Reference	Reference	Reference	Reference	Reference
Spring	−2.4 (−7.2–1.9)	−1.6 (−3.9–0.7)	−2.1 (−6.8–2.7)	6.6 ** (2.0–11.2)	5.5 (−1.6–12.6)	−7.1 (−14.7–0.6)	−1.1 (−4.3–2.0)
Summer	7.8 ** (2.5–13.2)	−0.2 (−2.9–2.5)	13.2 *** (8.5–17.8)	−0.2 (−5.2–4.8)	8.8 * (−7.9–4.8)	−15.3 *** (−23.6–−7.0)	5.5 *** (2.4–8.6)
Fall	2.9 (−1.7–7.6)	−1.2 (−3.6–1.1)	4.7 * (0.5–8.9)	3.3 (−1.1–7.8)	−1.5 (−5.0–4.3)	7.9 ** (2.2–13.6)	2.6 (−0.2–5.4)
	**Hookworms ^8^**	**Whipworms ^9^**	**Giant Roundworms ^10^**	***A. lumbricoides*** **^11^**	**Threadworms ^12^**	***S. stercoralis*** **^13^**	**Sanitation infrastructure deficiency ^14^**
N months	72	72	72	72	72	72	72
Sanitation-infrastructure-deficiency-related search terms ^12^	1.6 (−1.3–4.5)	2.7 (−2.1–7.5)	−1.9 (−4.6–0.7)	3.5 (−0.7–7.6)	3.0 (−1.4–7.4)	−1.8 (−6.9–3.3)	Not included
Year	−0.8 (−2.4–0.8)	−3.6 * (−6.6–−0.5)	−0.7 (−2.1–0.8)	−6.1 *** (−8.4–3.8)	−7.2 *** (−9.6–−4.8)	0.6 (−2.2–3.4)	4.6 *** (2.8–6.5)
Season							
Winter	Reference	Reference	Reference	Reference	Reference	Reference	Reference
Spring	0.7 (−3.7–5.1)	1.7 (−4.6–8.0)	0.3 (−3.7–4.3)	5.0 (−1.3–11.4)	−2.9 (−9.6–3.7)	5.4 (−2.4–13.1)	1.3 (−1.7–4.3)
Summer	8.0 *** (3.7–12.4)	−0.9 (−7.5–4.8)	−4.3 * (−8.2–−0.3)	−10.7 *** (−16.9–−4.5)	5.1 (−1.4–11.6)	2.1 (−5.4–9.7)	1.2 (−2.2–4.7)
Fall	1.6 (−1.3–4.5)	−1.4 (−7.5–4.8)	−1.5 (−5.1–2.1)	1.6 (−4.1–7.3)	0.4 (−1.4–7.4)	7.7 * (0.8–3.3)	0.2 (−2.8–3.2)

* = *p* < 0.05, ** = *p* < 0.01, *** = *p* < 0.001.

## Data Availability

Data analyzed are included as supplementary files.

## Supplementary Materials

The following supporting information can be downloaded at: https://www.mdpi.com/article/10.3390/tropicalmed8040212/s1, Supplementary File S1: Data used in analyses.
